# Novel restriction factor RNA-associated early-stage anti-viral factor (REAF) inhibits human and simian immunodeficiency viruses

**DOI:** 10.1186/1742-4690-10-S1-P57

**Published:** 2013-09-19

**Authors:** Kelly Marno, Babatunji Ogunkolade, Corinna Pade, Nidia Oliveira, Eithne O’Sullivan, Áine McKnight

**Affiliations:** 1 Centre for Immunology and Infectious Disease, Blizard Institute, Barts and The London School of Medicine and Dentistry, Queen Mary University of London, London, UK

## Background

The discovery of novel anti-viral restriction factors illuminates unknown aspects of innate sensing and immunity. We identified RNA-associated Early-stage Anti-viral Factor (REAF) using a whole genome siRNA screen for restriction factors to Human Immunodeficiency Virus (HIV) that act in the early phase of viral replication.

## Results

We observed more than 50 fold rescue of HIV-1 infection, using a focus forming unit (FFU) assay, following knockdown of REAF by specific siRNA. Quantitative PCR was used to measure the effect of REAF knockdown on two steps in the replication cycle - production of reverse transcripts and integration of viral cDNA. Both steps were strongly enhanced. Conversely, when REAF is over expressed in target cells fewer reverse transcripts are produced. Human REAF can also inhibit HIV-2 and simian immunodeficiency virus (SIV) infection. REAF associates with viral nucleic acid and may act to prevent reverse transcription.

## Conclusions

This report firmly places REAF alongside APOBECs and the recently described SAMHD1 as a potent inhibitor of HIV replication acting early in the replication cycle, just after cell entry. We propose that REAF is part of an anti-viral surveillance system destroying incoming retroviruses. This novel mechanism could apply to invasion of cells by any intracellular pathogen.

